# Physical stability and release properties of lumefantrine amorphous solid dispersion granules prepared by a simple solvent evaporation approach

**DOI:** 10.1016/j.ijpx.2020.100052

**Published:** 2020-07-16

**Authors:** Niraj S. Trasi, Sonal V. Bhujbal, Dmitry Y. Zemlyanov, Qi (Tony) Zhou, Lynne S. Taylor

**Affiliations:** a Department of Industrial and Physical Pharmacy, College of Pharmacy, Purdue University, West Lafayette, Indiana 47907, USA; b Birck Nanotechnology Center, Purdue University, West Lafayette, Indiana 47907, USA

**Keywords:** Lumefantrine, Amorphous solid dispersions, Salt formation, Physical stability, Release

## Abstract

Amorphous solid dispersions (ASDs) of lumefantrine, which has low aqueous solubility, have been shown to improve bioavailability relative to crystalline formulations. Herein, the crystallization tendency and release properties of a variety of lumefantrine ASD granules, formed on a blend of microcrystalline cellulose and anhydrous lactose, prepared using a simple solvent evaporation method, were evaluated. Several polymers, a majority of which contained acidic moieties, and different drug loadings were assessed. Crystallinity as a function of time following exposure to stress storage conditions of 40 °C and 75% relative humidity was monitored for the various dispersions. Release testing was performed and ASD characteristics were further evaluated using infrared and X-ray photoelectron spectroscopy (XPS). A large difference in stability to crystallization was observed between the various ASDs, most notably depending on polymer chemistry. This could be largely rationalized based on the extent of drug-polymer interactions, specifically the degree of lumefantrine-polymer salt formation, which could be readily assessed with XPS spectroscopy. Lumefantrine release from the ASDs also varied considerably, whereby the best polymer for promoting physical stability did not lead to the highest extent of drug release. Several formulations led to concentrations above the amorphous solubility of lumefantrine, with the formation of nano-sized drug-rich aggregates. A balance between the ability of a given polymer to promote physical stability and drug release may need to be sought.

## Graphical abstract

**Figure:**
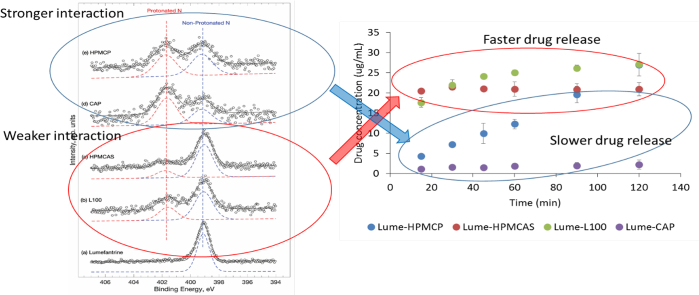

## 1 Introduction

Amorphous solid dispersions (ASDs) are increasingly being utilized to improve the dissolution and apparent solubility of poorly water soluble drugs (Hancock and Zografi, 1997; Newman et al., 2015; Prasad et al., 2014; Shah et al., 2013). The reasons behind the improved in vivo performance of ASDs include the use of a high energy form of the drug, and enhanced release properties when mixed with a hydrophilic polymer. One risk associated with using an amorphous solid dispersion, however, is that the amorphous drug reverts back to the more stable crystalline form (Aso et al., 2000; Caron et al., 2010; Yoshioka et al., 1994), especially in the presence of water (Guo et al., 2000; Ohtake and Shalaev, 2013). To prevent or significantly delay the transformation to the crystalline form, the preparation of ASDs requires the use of stabilizers such as vinyl or cellulosic polymers (Law et al., 2001; Six et al., 2004; Sotthivirat et al., 2013). It is desirable that these polymers are miscible with the active pharmaceutical ingredient (API) (Qian et al., 2010), with the formation of drug-polymer interactions (Kothari et al., 2015). Additionally, polymers with a high glass transition temperature (T_g_) can increase the T_g_ of the resultant dispersion to a value above that of the pure drug, lowering the molecular mobility at the storage temperature (Van den Mooter et al., 2001). Mixing of the polymer with the amorphous drug also decreases the drug chemical potential and thus the thermodynamic driving force for crystallization by a dilution effect. It should be noted, however, that depending on the drug-polymer ratio and the method of preparation, phase separation can still occur with the formation of separate amorphous drug and polymer-rich regions which can result in crystallization in the drug-rich domains (Purohit and Taylor, 2015).

Amorphous dispersions can be prepared using processes including spray drying, melt extrusion, freeze-drying, milling, and solvent impregnation, with the first two processes being the most commonly used for large scale manufacturing especially for non-sterile products (Friesen et al., 2008; Repka et al., 2008). Each of these processes has advantages and disadvantages with a common problem being the high initial cost of the equipment. There is, therefore, an interest in developing and evaluating the viability of methods to manufacture amorphous dispersions that are cheaper and that use simpler equipment, such as wet granulation or anti-solvent precipitation. These methods potentially could be used for manufacture in settings where a large capital investment in highly specialized equipment is not possible. In particular, therapies used to mitigate infectious diseases, which are widely employed in low-income countries, could benefit from more economical manufacturing approaches, that lead to formulations with enhanced in vivo exposure. Preparation of ASDs using these alternative processes will differ from conventional methods due to the slow solvent evaporation during granulation and the exposure of the drug-polymer system to an aqueous environment during anti-solvent precipitation, and are therefore likely to be only useful for drugs with certain physicochemical properties, namely those with a low tendency to crystallize during the manufacturing operation. It is therefore of interest to identify drugs used to treat infectious diseases that might be amenable to manufacturing using these alternative approaches.

Malaria is an extremely prevalent infectious disease that is transmitted through mosquito bites and is caused by the Plasmodium protozoal parasite. There are five species of the parasite of which one (*P. falciparum*) is the most deadly (Cox, 2010). There is no vaccine currently available to prevent malaria and it is treated using a combination of medications usually containing an artemisinin (obtained from the plant *Artemisia annua*) derivative. Coartem® (manufactured by Novartis) is one of the five artemisinin based drug combinations (others being dihydroartemesinin-piperaquine, artesunate-mefloquine, artesunate-sulfadioxine-pyrimethamine and artesunate-amodiaquine) that is used for the treatment of malaria and is the only combination that is approved for use in the United States (Tan and Arguin, 2017). Coartem® is a fixed dose combination of 20 mg artemether and 120 mg lumefantrine with both drugs present in their crystalline solid form. Artemether is the fast acting component which reduces the parasite mass, relieving symptoms while lumefantrine is the long-acting drug that prevents recrudescence and the development of resistance in the microbe. While artemether has poor water solubility, it appears to have acceptable bioavailability when given orally (Teja-Isavadharm et al., 1996). However, the absorption of lumefantrine is very variable and changes with food; it is recommended that the medication be taken after meals since the absorption is highly dependent on food intake (Borrmann et al., 2010). Furthermore, there have been reports of treatment failures, attributed to poor absorption and low blood concentrations of lumefantrine either due to administration on an empty stomach or other intestinal absorption issues (Mizuno et al., 2009; Repetto et al., 2011). Given that the symptoms of malaria include nausea, vomiting, fever, etc., the patient may not be ingesting sufficient food while infected, and in poverty stricken areas the patients may not have access to enough food (Wernsdorfer, 2004). Consequently, solubility enhancement strategies, especially if they avoid the need for administration with food, are of interest.

Various approaches have been employed to try and improve the solubility and thus anti-malarial activity of both of the components in Coartem® including nanostructured lipid carriers, self-emulsified solid dispersions, solid lipid microparticles and nanoemulsions (Agbo et al., 2016; Laxmi et al., 2015; Parashar et al., 2016; Tayyab Ansari et al., 2016). Recently, it was demonstrated that lumefantrine amorphous solid dispersions improved its oral absorption relative to the crystalline reference (Jain et al., 2017). Given that lumefantrine amorphous solid dispersions appear to be a viable approach to improve in vivo exposure, it was of interest to further investigate these systems in the context of manufacturing methods, and the characteristics of the resultant dispersions. Thus, the goal of the current study was twofold. First to determine if simple, low cost, manufacturing approaches could be used to produce lumefantrine amorphous solid dispersions. Second, to characterize the phase behavior of the ASDs during storage and release in order to better understand the origin of the improved absorption previously reported, as well as to evaluate the robustness of the ASDs to crystallization following exposure to accelerated stability conditions.

## 2 Materials

**Fig. 1:**
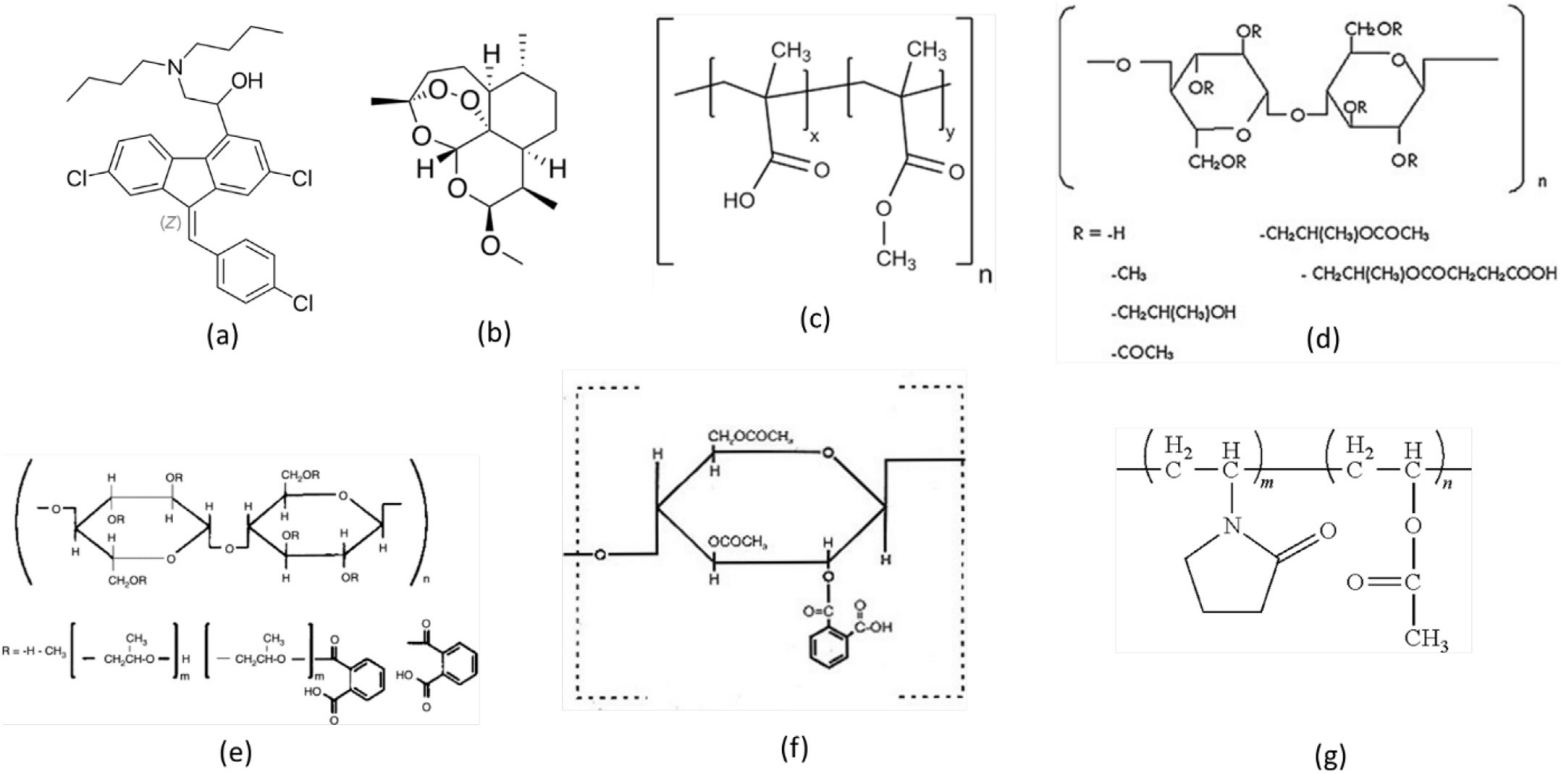
Chemical structures of (a) lumefantrine, (b) artemether (c) Eudragit L100, (d) HPMCAS, (e) HPMCP, (f) CAP and (g) PVPVA.

Lumefantrine and artemether were obtained from Euroasia, India. Prodan, pyrene and cellulose acetate phthalate (CAP), microcrystalline cellulose (MCC) and anhydrous lactose were purchased from Sigma-Aldrich (St. Louis, MO). Eudragit L100 was obtained from Degussa Rohm Pharma Polymers (Darmstadt, Germany), hydroxypropyl methylcellulose phthalate (HPMCP-50) and hydroxypropyl methylcellulose acetate succinate (HPMCAS-MF) were supplied by Shin-Etsu Chemicals (Tokyo, Japan) and poly*v*inylpyrrolidone-vinyl acetate 64 (PVPVA) was sourced from BASF (Ludwigshafen, Germany). Dichloromethane (DCM), methanol (MeOH) and ethanol (EtOH) were obtained from Fisher-Scientific (Pittsburg, PA). The chemical structure of model drugs and polymers are shown in Fig. 1.

## 3 Methods

### 3.1 Preparation of dispersions and granules

6:4 and 4:6 *w*/w lumefantrine:polymer dispersions were made by dissolving the drug and polymer in an organic solvent combination and then rotary evaporated using a Buchi Rotavapor-R (Buchi Corp, New Castle, DE) attached to a water bath (BM200, Yamato Scientific America, Santa Clara, CA) kept at 40 °C. The solvents used to make the dispersions of the drug were 8:2 v/v DCM:MeOH for CAP and HPMCP, DCM only for PVPVA, 1:1 DCM:MeOH for HPMCAS while Eudragit L100 was dissolved in 1:1 DCM:EtOH.

Solvent granulation was performed by dissolving 1080 mg of the polymer in 10 mL of the appropriate organic solvent system. To this was added 720 mg of lumefantrine to give a 4:6 w/w ratio of lumefantrine:polymer. This solution was added slowly to 3.6 g of 1:1 w/w ratio of MCC:anhydrous lactose and mixed with a spatula while allowing the solvent to evaporate. This was then placed under vacuum overnight to ensure complete removal of the organic solvents and then cryomilled (6750 freezer mill, Spex Sampleprep, Metuchen, NJ).

### 3.2 Differential scanning calorimetry (DSC)

Thermal analysis of the pure materials as well as the rotary evaporated samples was performed in a DSC Q2000 (TA Instruments, New Castle, DE) cooled with a RCS 90 setup. The instrument was calibrated for temperature and enthalpy using indium and tin. Dry nitrogen at 50 mL/min was used as the purge gas. The crystalline drug was heated to a temperature of 150 °C (above its melting point) at a rate of 10 °C/min, cooled to −30 °C and then reheated at 10 °C/min to determine the T_g_.

The rotary evaporated dispersions were analyzed by modulated DSC by first cooling to −10 °C and then heating to 150 °C at a heating rate of 2 °C/min and a modulation of 1 °C every 60s. The glass transition temperature (T_g_) was determined using reversible heat flow using the Universal Analysis 2000 software (TA Instruments, New Castle, DE).

### 3.3 Fourier transform infra-red spectroscopy (FTIR)

FTIR analysis was performed using a Vertex 70 IR Spectrophotometer (Bruker Optics, Billerica, MA.). A total of 64 scans were averaged in the spectral range of 400–4000 cm^−1^ and the powder spectra were obtained using a Golden Gate attenuated total reflectance (ATR) accessory (Specac, Fort Washington, PA). The ATR unit, as well as the detector compartment, were kept continuously flushed with dry air and a background scan was taken before recording the sample spectra.

### 3.4 Dissolution

Dissolution of granulated lumefantrine was performed in 100 mL of pH 6.8 phosphate buffer 10 mM maintained at 37 °C using a jacketed beaker and stirred at 150 rpm using a magnetic stirrer. This pH was selected since a majority of the polymers evaluated are insoluble in acidic media. Granulated material, containing around 20 mg lumefantrine, was compressed into a pellet with 10% croscarmellose sodium as a disintegrant and added to the dissolution medium. Samples were filtered using 1 μm glass syringe filters and the filtrate was analyzed by UV spectroscopy using a 1 cm pathlength ultraviolet (UV) dip probe coupled to a spectrometer (S.I. Photonics, Tucson, AZ). The filtrate was returned to the dissolution medium after analysis. A wavelength of 310 nm was used to quantify lumefantrine. A calibration curve was made using a stock solution of lumefantrine prepared by first dissolving 10 mg of drug in 100 μL of chloroform and then diluted to 10 mL with acetonitrile which was further diluted with acetonitrile to different concentrations ranging from 1 to 25 μg/mL and analyzed with the dip probe. The calibration curve had a R^2^ value of 0.999.

### 3.5 Fluorescence spectroscopy

Fluorescence spectroscopy was performed using the Shimadzu RF 5301 PC spectroflurophotometer (Kyoto, Japan). 1 μg/mL prodan and 0.2 μg/mL pyrene were used as fluorescent probes to evaluate the possible formation of amorphous nanodroplets of lumefantrine. For prodan, the excitation wavelength was 370 nm and the emission was measured between 400 and 600 nm. For pyrene, the excitation wavelength was 332 nm and the emission range was between 350 and 450 nm. The excitation and emission slits were kept at 10 and 1.5 nm for the experiments.

A lumefantrine stock solution of 1 mg/mL was prepared in dimethyl acetamide and added in increasing amounts to 10 mL of 50 mM pH 6.8 phosphate buffer containing 0.2 μg/mL pyrene covering a lumefantrine concentration from 0.5μg/mL to 12 μg/mL. The experiment was repeated twice and the mean value plotted. This experiment enabled the amorphous solubility of lumefantrine to be approximated.

Fluorescence analysis was also performed on the dissolution media (containing 0.2 μg/mL pyrene) following dissolution of the dispersion granules. In this case, the samples were filtered using 0.45 μm PTFE syringe filters, since these did not absorb the fluorescent probes, whereas adsorption was observed for glass filters.

### 3.6 Particle size and zeta potential

The particle size and zeta potential of the particles in the filtrate obtained by passing the dissolution media through a 0.22 μm cellulose acetate syringe filter following dissolution of the granules were determined using a Zetasizer Nano-ZS (Malvern Instruments, Westborough, MA). The sample was placed in a plastic cuvette and the particle size was determined using the principle of dynamic light scattering (DLS) using a backscatter detector at an angle of 173°. The zeta potential was measured by the instrument using micro-electrophoresis and electrophoretic light scattering technology. An average of three experiments is reported.

### 3.7 X-ray powder diffraction (XRPD)

A Rigaku Smartlab™ diffractometer (Rigaku Americas, Texas, USA) with a Cu-Kα radiation source and a D/tex ultra detector was used to determine the x-ray diffraction profiles of the powders. Samples were prepared on glass sample holders and powder patterns were obtained from 4 to 40° 2θ at a scan speed of 4°/min and a step size of 0.02°. The voltage and current used were 40 kV and 44 mA respectively.

### 3.8 X*-*Ray photoelectron spectroscopy

XPS spectra were collected using a Kratos Axis Ultra DLD spectrometer using monochromic Al Kα radiation (1486.6 eV) at constant pass energy (PE) of 20 and 160 eV for high-resolution and survey spectra, respectively. A build-in Kratos charge neutralizer was used to avoid non-homogeneous electric charge of non-conducting powder samples and to achieve better resolution. The charge correction was performed for each acquisition point setting the C—C component of the C 1 s peak to a binding energy of 284.8 eV. Binding energy (BE) values refer to the Fermi edge and the energy scale was calibrated using Au 4f_7/2_ at 84.0 eV and Cu 2p_3/2_ at 932.67 eV. The powder samples were placed on a stainless steel sample holder bar using a double-sided sticking Cu tape. XPS data were analyzed with CasaXPS software (www.casaxps.com). Curve-fitting was performed following a Shirley background subtraction using Gaussian/Lorentzian peak shapes. The atomic concentrations of the elements in the near-surface region were estimated after a Shirley background subtraction taking into account the corresponding Scofield atomic sensitivity factors and inelastic mean free path (IMFP) of photoelectrons using standard procedures in the CasaXPS software assuming homogeneous mixture of the elements within the information depths (~10 nm).

## 4 Results

### 4.1 Dispersion crystallinity

**Fig. 2:**
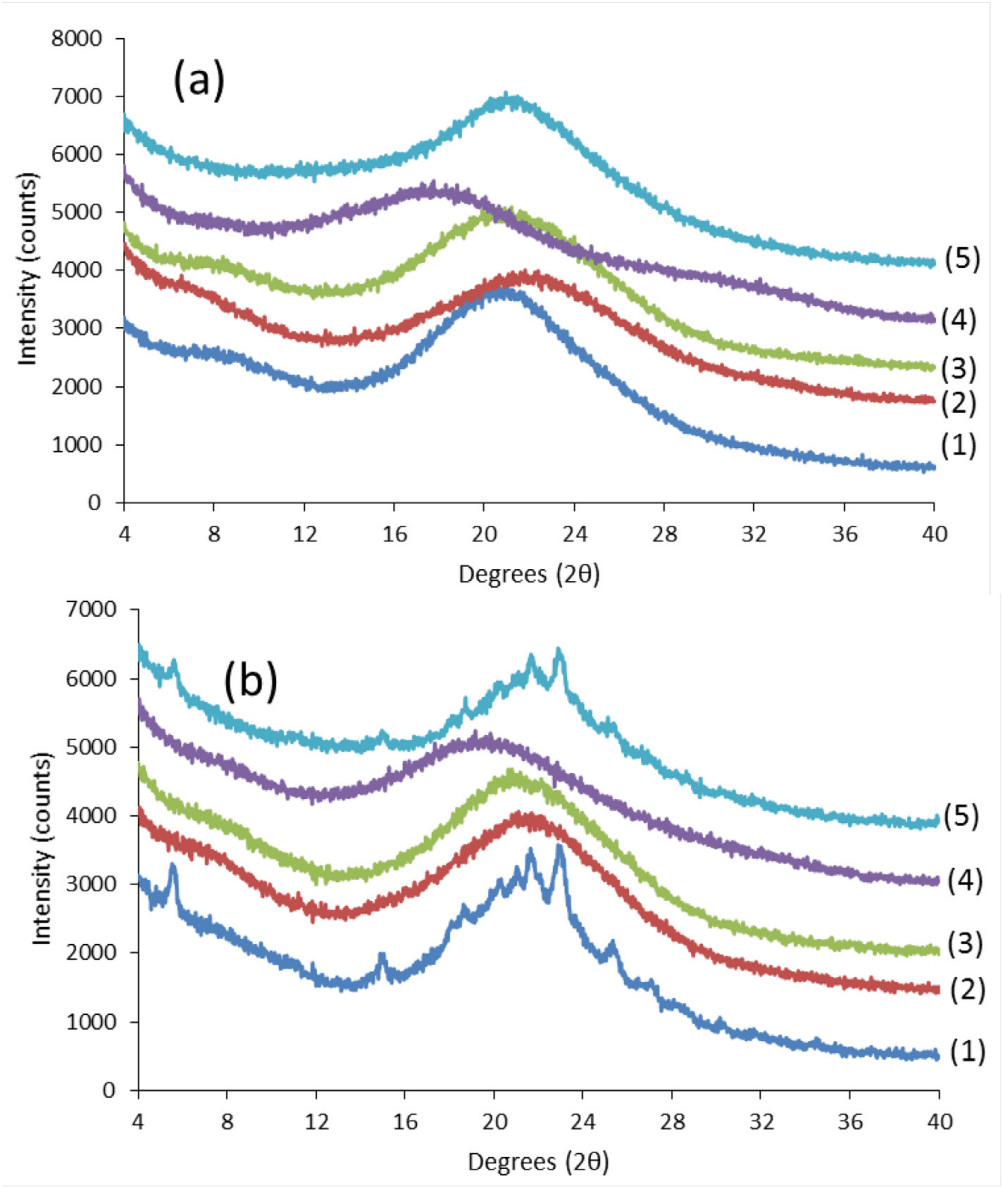
X-ray diffraction patterns of (a) 4:6 Lumefantrine:polymer and (b) 6:4 lumefantrine:polymer prepared by rotary evaporation of lumefantrine with (1) HPMCAS, (2) CAP, (3) HPMCP, (4) Eudragit L100, (5) PVPVA.

**Fig. 3:**
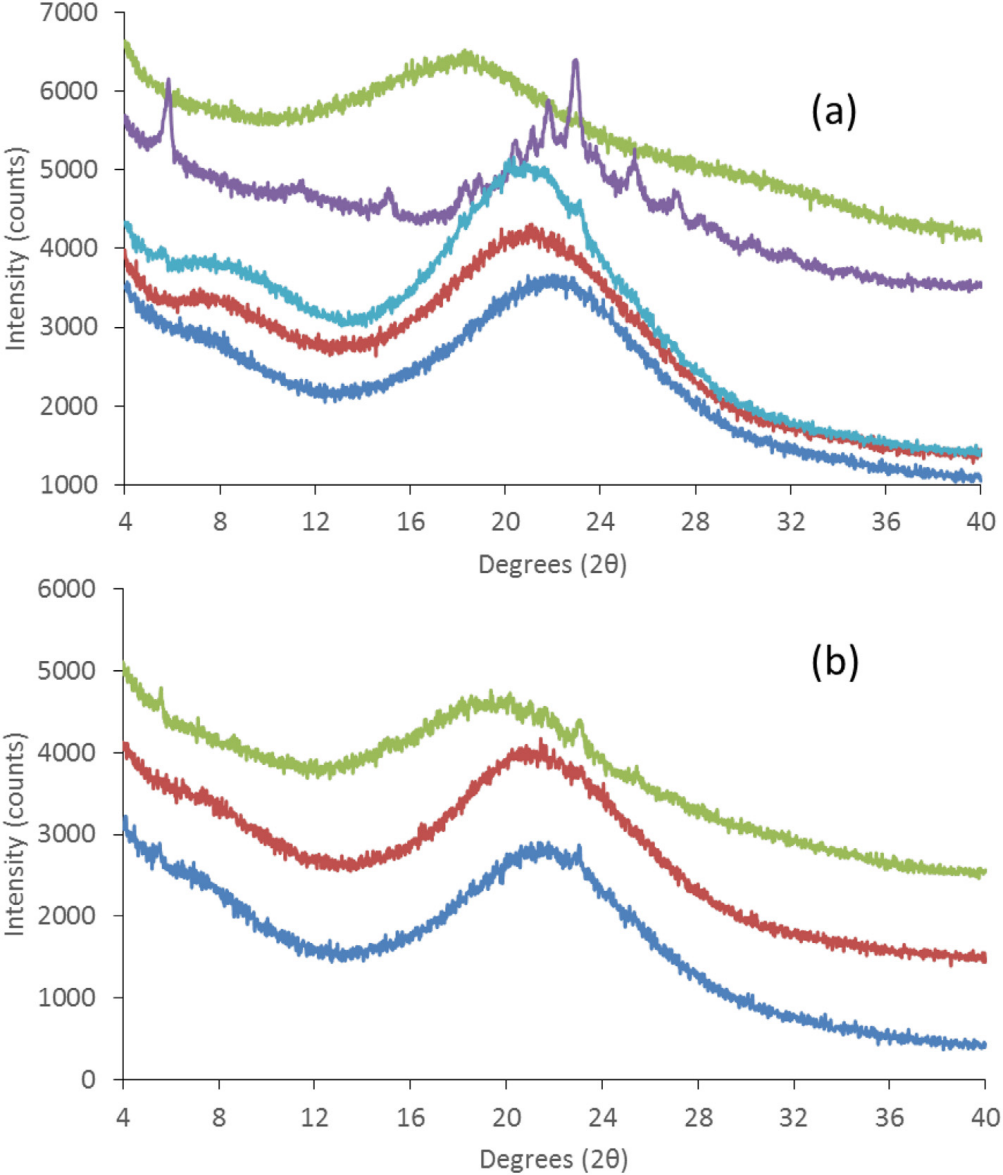
(a) XRPD of the 4:6 Lume:polymer dispersions upon storage at 40C/75%RH. The traces from bottom to top are Lume:CAP (3 months storage), Lume:HPMCP (3 months storage), Lume:HPMCAS (4 weeks storage), Lume:PVPVA (1 week storage) and Lume:L100 (3 months storage) and (b) XRPD of the 6:4 Lume:polymer dispersions upon storage at 40C/75%RH. The traces from bottom to top are Lume:CAP (2 weeks storage), Lume:HPMCP (6 weeks storage), Lume:L100 (2 weeks storage),

The X-ray powder diffraction patterns of the dispersions immediately after preparation showed that all the dispersions at 4:6 drug:polymer ratio were X-ray amorphous (Fig. 2a). The 6:4 dispersions on the other hand showed crystalline peaks for HPMCAS and PVPVA systems indicating that these polymers were not effective at inhibiting the drug crystallization during the solvent evaporation process, for the higher drug loading (Fig. 2b). When the dispersions were kept under accelerated storage conditions of 40 °C/75% RH open dish conditions (Fig. 3), it was noted that the drug in the 4:6 Lume:PVPVA dispersion crystallized within one week while the 4:6 dispersions of HPMCP, L100 and CAP were found to be stable under these conditions for up to 3 months. The HPMCAS dispersion showed small peaks after 1 month of storage. Small peaks were also visible in the 6:4 CAP and L100 systems after 2 weeks at 40 °C/75% RH while it took 6 weeks for peaks to be discernable in the 6:4 HPMCP dispersion. Upon continued storage, these peaks grew faster in the L100 dispersion relative to CAP while in the HPMCP dispersion the peak remained very small with little to no increase in the peak area after 3 months (data not shown). In summary, the most stable systems were the 4:6 CAP, L100, and HPMCP dispersions.

### 4.2 Thermal analysis

**Fig. 4:**
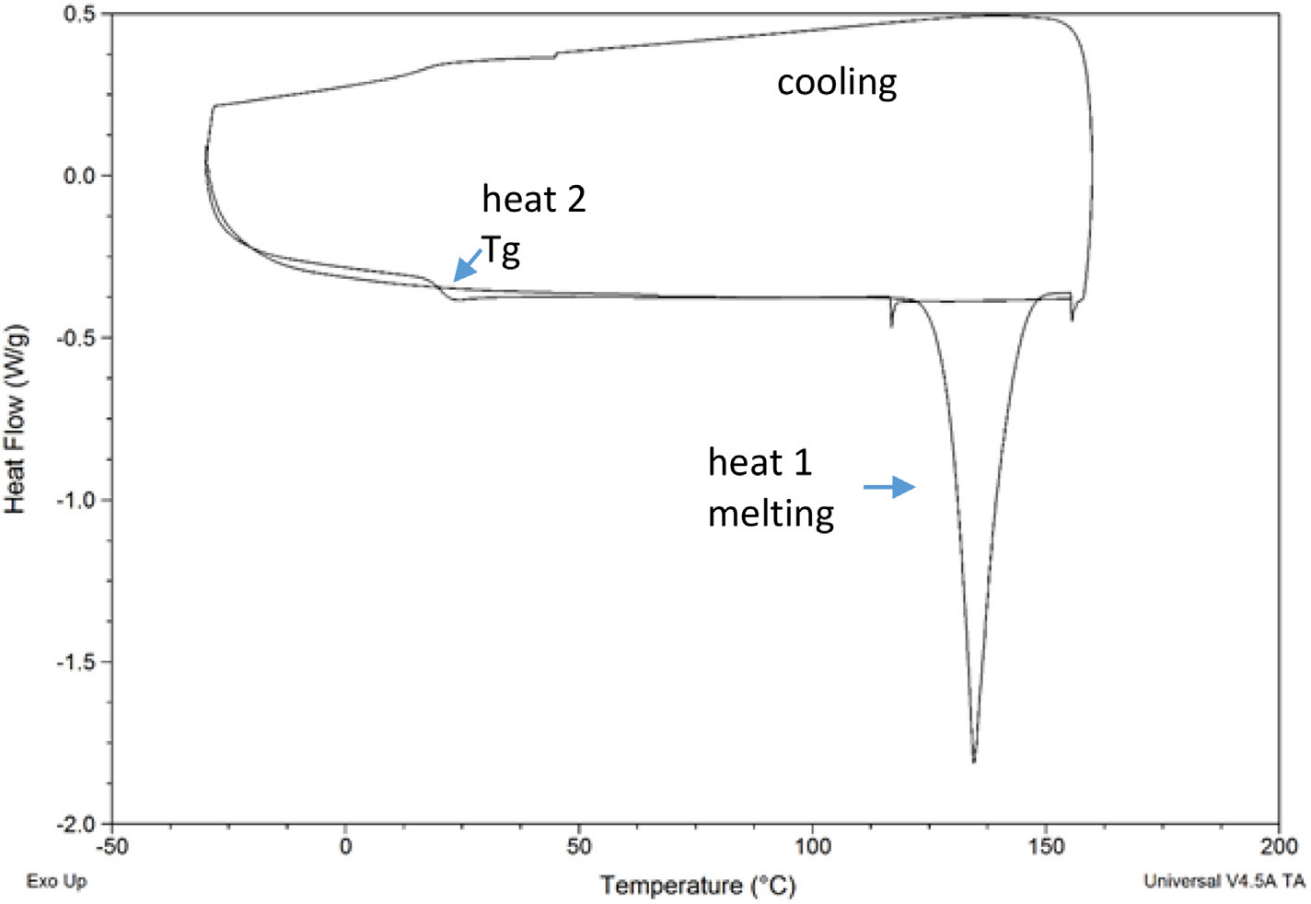
DSC thermogram crystalline lumefantrine showing a melting endotherm and a glass transition event at around 18 °C.

Based on the results of DSC analysis (Fig. 4), lumefantrine was found to exhibit class 3 behavior (Baird et al., 2010), where the compound has good glass forming ability and good glass stability. In other words, it could be readily converted into an amorphous material upon melt quenching and resisted devitrification upon reheating. The onset melting temperature of the crystalline material was 129 °C and the Tg (onset) was 18 (±1)°C. Artemether, on the other hand, demonstrated a class 1 behavior wherein the compound, which melted at the relatively low temperature of 87 °C, crystallized upon cooling at relatively low extent of undercooling, with recrystallization commencing around 60 °C (Fig. S1).

Attempts to make amorphous dispersions of artemether with various polymers were unsuccessful due to partial crystallization, and were therefore not studied further. The T_g_s of the various dispersions of lumefantrine are summarized in Table 1. The 6:4 Lume:HPMCAS and PVPVA dispersions were not analyzed since the drug crystallized during preparation. The L100 and CAP dispersions exhibited a small melting endotherm suggesting some recrystallization during heating in the DSC given that the starting materials were X-ray amorphous. No melting was observed for the corresponding 4:6 dispersions indicating that the drug was better stabilized at this drug loading with these polymers. However, the 4:6 Lume:HPMCAS and PVPVA dispersions showed some recrystallization during the heating step. No clear T_g_ could be identified for the PVPVA dispersion. The L100 dispersions were highly variable in terms of the T_g_ measurements. The most effective polymer at preventing lumefantrine crystallization appeared to be HPMCP since no recrystallization was observed even at 60% drug loading. The thermal analysis results are in broad agreement with the XRPD results in terms of identifying the more stable dispersions i.e. those that did not show signs of crystals in the DSC experiment, namely the 4:6 Lume:polymer dispersions with HPMCP, CAP, and L100. Example thermograms are shown in Fig. S2.

**Table 1:** Onset glass transition temperatures (Tg) of the pure drug and polymers and of the 6:4 and 4:6 dispersions prepared by rotary evaporation. (*n* = 3, standard deviations shown in parentheses).

Sample	Tg (°C)	Tg (°C)	Tg (°C)
		6:4 Lume:polymers	4:6 Lume:polymers
Lumefantrine	18 (1)		
HPMCP	138 (0)	85 (0)	113 (0)
Eudragit L100	194 (0)	Crystallized	163 (28)
CAP	161 (2)	87(1)^a^	116 (1)
HPMCAS	117 (1)	Crystallized	76 (1)^a^
PVPVA	101 (2)	Crystallized	No clear Tg^a^

a Evidence of melting was observed following heating above the Tg indicating recrystallization during the DSC experiment.

### 4.3 FTIR analysis

**Fig. 5:**
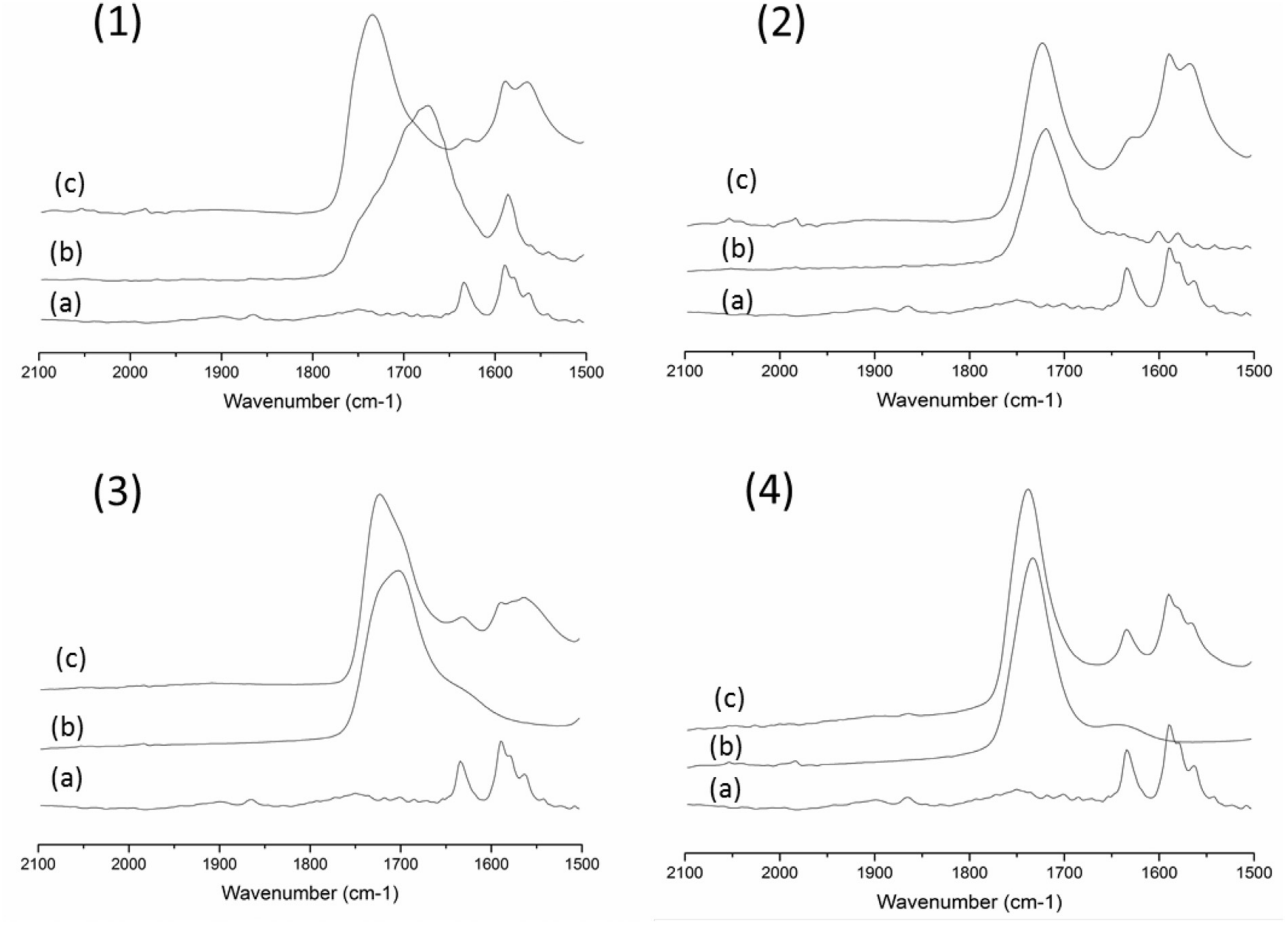
FTIR spectra of the carbonyl region of (a) amorphous lumefantrine, (b) pure polymer and (c) 4:6 Lume:polymer ASD containing (1) CAP, (2) HPMCP, (3) Eudragit L100 and (4) HPMCAS.

Infrared spectra of amorphous lumefantrine, neat polymers and the 4:6 dispersions are summarized in Fig. 5, and the spectrum of crystalline lumefantrine is shown in Fig. S3. Peak shifts to higher wavenumbers are visible in the carbonyl region of all the dispersions prepared with the acidic polymers. Only the polymers contain carbonyl groups, and these groups are anticipated to form self‑hydrogen bonds with the carboxylic acid donor present in the polymer. The shift of the polymer carbonyls to higher wavenumbers in the presence of lumefantrine suggests a change in H-bonding in the presence of the drug. The biggest shift is observed for the lume:CAP dispersion in which the carbonyl peak moves from 1677 cm^−1^ for the pure polymer to 1732 cm^−1^ in the drug:CAP dispersion. The other dispersion with a significant change in the carbonyl region is the Eudragit L100 system which has a peak at 1701 cm^−1^ with a shoulder at 1723 cm^−1^, and in the presence of lumefantrine now shows a peak 1723 cm^−1^ with a shoulder at 1701 cm^−1^. For the 4:6 lumefantrine-HPMCP dispersion there is a slight shift in the peak position of the carbonyl group from 1718 cm^−1^ in the pure polymer to 1722 cm^−1^ indicating some interaction between the drug and polymer. HPMCAS also shows a small shift in the carbonyl peak from 1733 cm^−1^ to 1737 cm^−1^ when made into a dispersion with lumefantrine. In addition, we note a new peak at around 1560 cm^−1^ for CAP, HPMCP, and L100 dispersions. This is likely due to a carboxylate vibration, suggesting that some level of proton transfer may have occurred between the drug and polymer, converting some portion of the carboxylic acid groups into carboxylate groups.

The region of the spectrum that has vibrations from the acidic, donor groups of the polymers showed broad peaks, and inferences about intermolecular interactions could not be made.

### 4.4 X-ray photoelectron spectroscopy

**Fig. 6:**
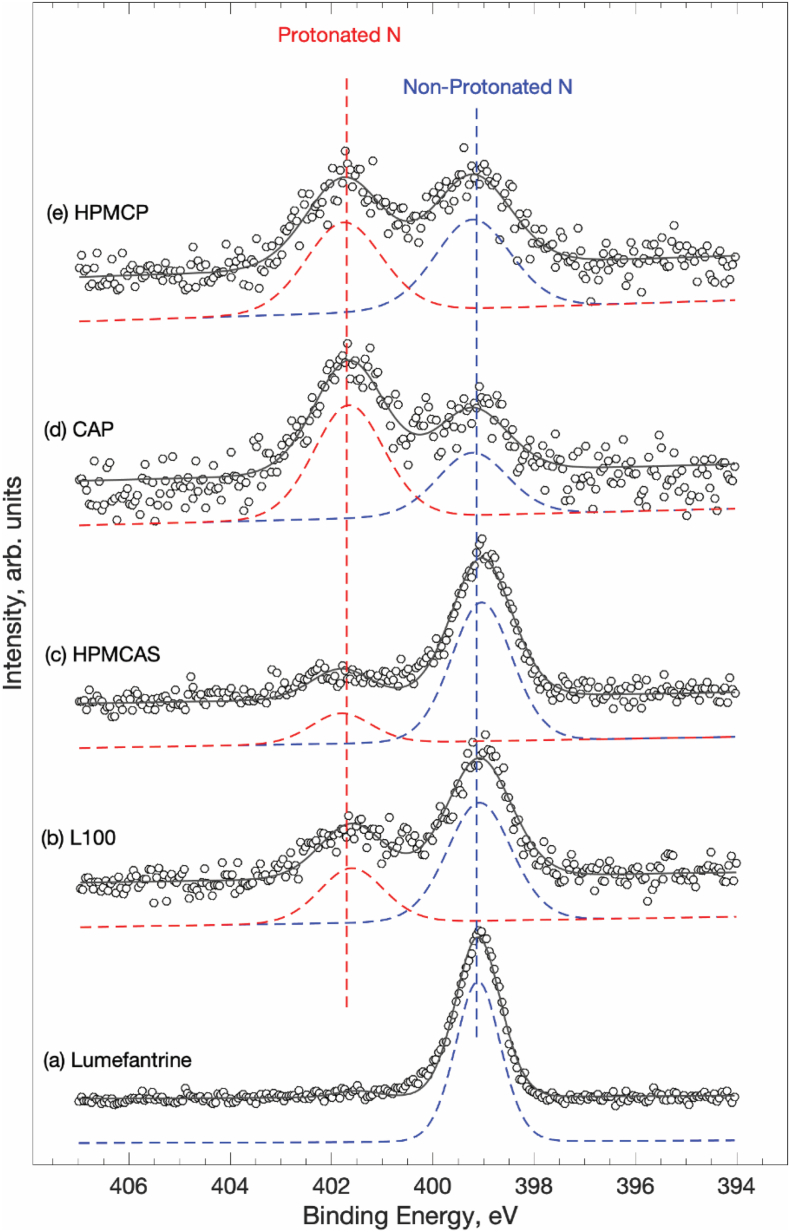
XPS spectra of lumefantrine ASDs. From bottom to top, lumefantrine, L100, HPMCAS, CAP, HPMCP.

The extent of protonation of lumefantrine in amorphous solid dispersions containing a 4:6 ratio of drug:polymer was evaluated using X-ray photoelectron spectroscopy. As shown in Fig. 6, lumefantrine shows a single N 1 s peak at 399 eV. None of the acidic polymers contain nitrogen, and therefore show no peaks that interfere with the drug N 1 s peak. In the presence of the acidic polymers, a second component of the N 1 s peak at 402 eV emerges. This peak has been previously assigned to a protonated nitrogen in lumefantrine (Song et al., 2016), whereby the increase in binding energy is consistent with the increased energy required to remove the electrons in the presence of a positive charge. Thus, the basic lumefantrine undergoes partial salt formation with the acidic polymers. However, the extent of protonation is clearly different for the various polymers, with HPMCAS showing only a small amount of protonation, while CAP shows the largest extent. Table 2 shows the percentage protonation, as calculated from peak fitting of the XPS data, for the various dispersions.

**Table 2:** Percentage protonation of lumefantrine in the presence of different acidic polymers. The samples analyzed were ASDs with a 4:6 drug:polymer ratio.

ASD	% Protonation
Lumefantrine	7 (5)
Lume-HPMCAS	16 (2)
Lume-L100	33 (2)
Lume-HPMCP	48 (2)
Lume-CAP	61 (3)

### 4.5 Fluorescence spectroscopy

The amorphous solubility can be determined from the concentration where phase separation occurs in an aqueous solution, with the formation of a colloidal, drug-rich phase and a water-rich phase. Because, the drug-rich phase is disordered and less polar than bulk aqueous solution, other molecules may mix with this phase. When the fluorescent probe, pyrene, partitions into the drug rich nano-droplet phase formed when the drug amorphous solubility is exceeded, a decrease in the local environmental polarity results in a change in the pyrene emission spectrum (Glushko et al., 1981).

**Fig. 7:**
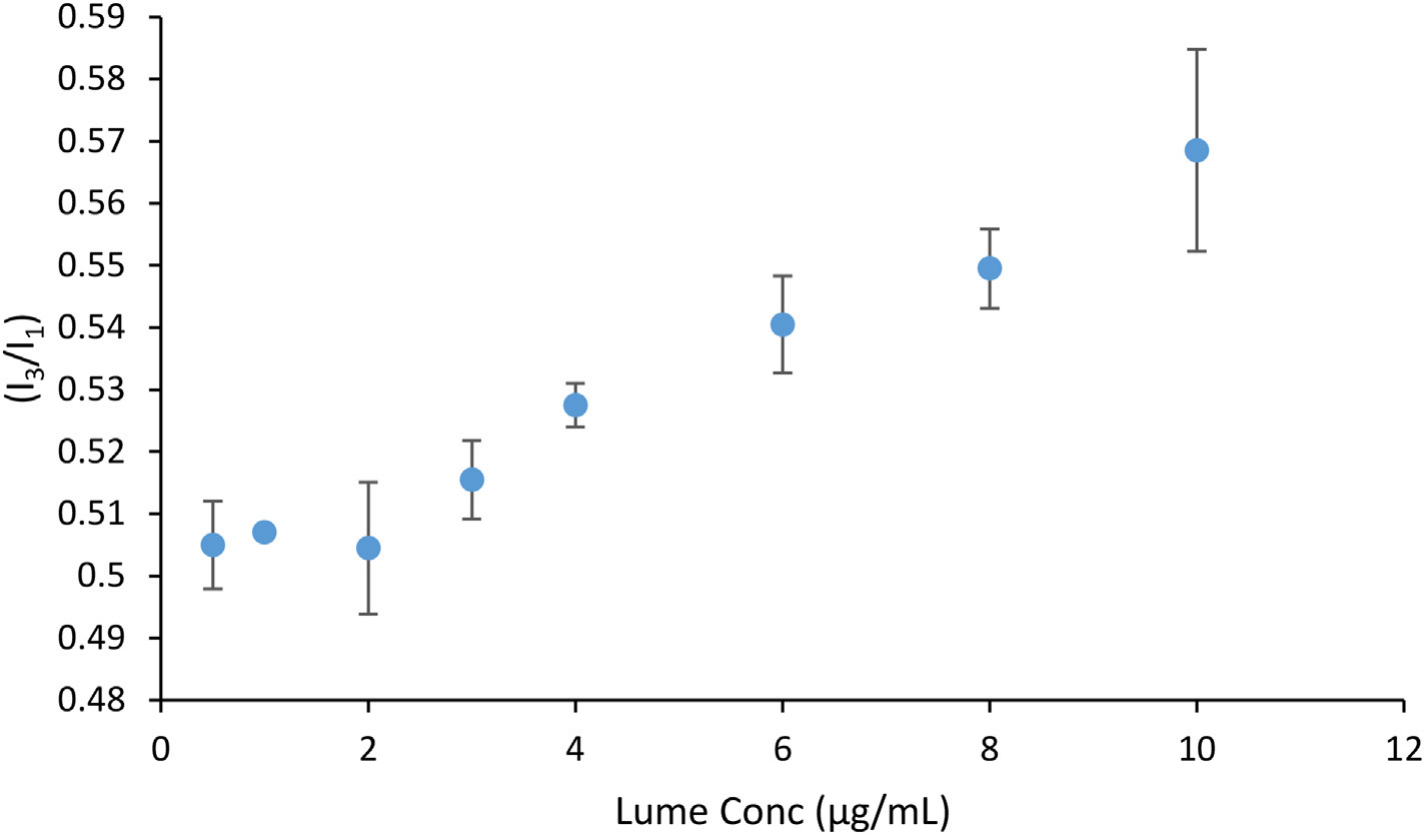
Plot of the ratio of I_3_/I_1_ of pyrene 0.2 μg/mL upon addition of increasing amount of lumefantrine stock solution. A deflection in the plot is seen between 2 and 3 μg/mL. Error bars indicate range, *n* = 2.

Fig. 7 shows the change in the ratio of the pyrene peaks I_3_/I_1_ upon serial addition of the drug into an aqueous solution containing pyrene. An abrupt change in slope occurs when the drug concentration exceeds 2 μg/mL. Beyond this concentration, the ratio increases, which can be explained by more and more probe partitioning into the increasing quantity of amorphous nanodroplets. These results indicate that the amorphous solubility of the drug is between 2 and 3 μg/mL in 50 mM pH 6.8 phosphate buffer.

### 4.6 Release studies

**Fig. 8:**
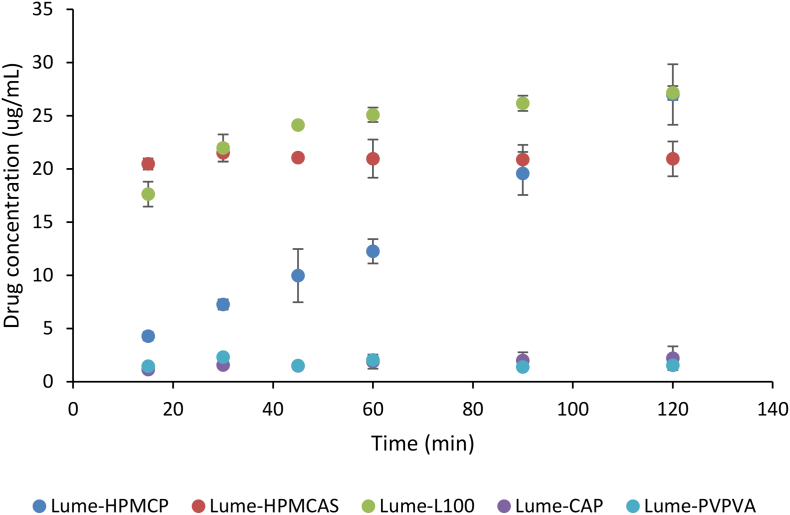
Dissolution profile of 4:6 lume:polymer granules in 100 mL of 50 mM pH 6.8 buffer.

The concentrations released during non-sink dissolution of the granulated powders, and after filtration using a 1 μm glass syringe filter are shown for different time points in Fig. 8. The fastest release was observed for the L100 dispersion granules with a final concentration of 28 μg/mL after 2 h followed by the HPMCAS granules which achieved a final concentration of 21 μg/mL in solution at 2 h. The HPMCP granules dissolved more slowly but steadily, resulting in a higher final concentration (27 μg/mL) than the HPMCAS granules. PVPVA granules dissolved poorly while the granules made with CAP released the least amount of drug, resulting in a final concentration of only 1.5 μg/mL, not even achieving the amorphous solubility of the drug.

For some of the systems, the concentrations obtained were significantly higher than the amorphous solubility of lumefantrine, suggesting that colloidal drug species were present. These species have been shown to form following dissolution of some ASDs (Harmon et al., 2016; Jackson et al., 2016).

To characterize these species, the filtered solutions, obtained after dissolution for 2 h, were analyzed by DLS to determine particle size, and separate measurements were conducted to evaluate the zeta potential with results shown in Table 3. Scattering species were observed following release from L100, HPMCAS and HPMCP dispersions. The size of colloidal species were found to be small, ranging from 110 to 180 nm depending on the polymer used to form the dispersion. The charge on the particles was also quite high, most likely due to the presence of the ionized polymer and/or drug at the particle-water interface. The CAP dispersion had a very low count rate and therefore the data was not considered.

**Table 3:** Particle size and zeta potential as determined using DLS after dissolving 4:6 lumefantrine granules in 50 mM pH 6.8 PO_4_ buffer. The value inside the parenthesis is the standard deviation, *n* = 3.

ASD	Zavg (nm)	Zeta potential (mV)
Lume-L100	116 (16)	−27 (2.6)
Lume-HPMCAS	179 (15)	−20 (1)
Lume-HPMCP	116 (6)	−22 (3)

**Fig. 9:**
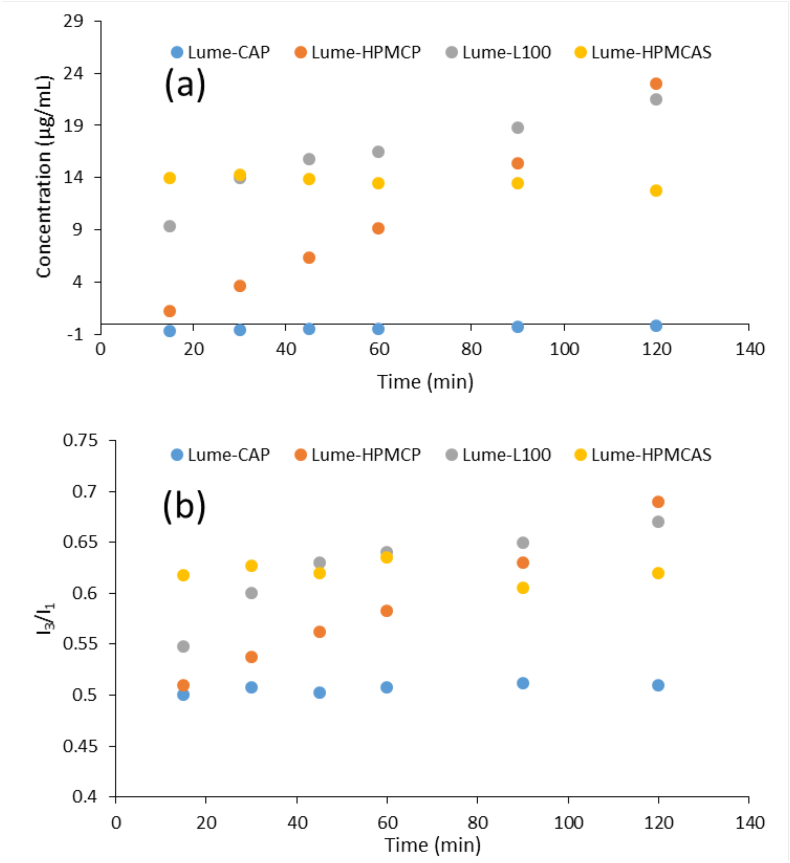
(a) Dissolution profile of lumefantrine-polymer granules in 50 mM pH 6.8 phosphate buffer and (b) Ratio of pyrene emission peaks of the filtered dissolution samples.

While the DLS results confirm the presence of submicron species, additional studies are necessary to determine if the drug-rich colloids are indeed amorphous in nature, as expected if they form via the process of LLPS. Hence, additional release studies were carried out in the presence of 0.2 μg/mL pyrene, where the expectation is that the fluorescence spectrum of the probe, as assessed from the I_3_/I_1_ ratio, will change if amorphous drug aggregates form as described previously (Jackson et al., 2016). When the drug concentration in solution during dissolution is compared to the change in the peak ratio of the pyrene emission spectrum, it can be observed that there is a relationship between these two parameters (Fig. 9). In other words, granules that lead to solution concentrations above the amorphous solubility result in higher I_3_/I_1_ ratios. This confirms that the high drug concentration observed during dissolution results in the formation of amorphous nanodroplets which pass through the 0.45 μm PTFE syringe filter.

### 4.7 Additional dissolution studies on CAP dispersions

**Fig. 10:**
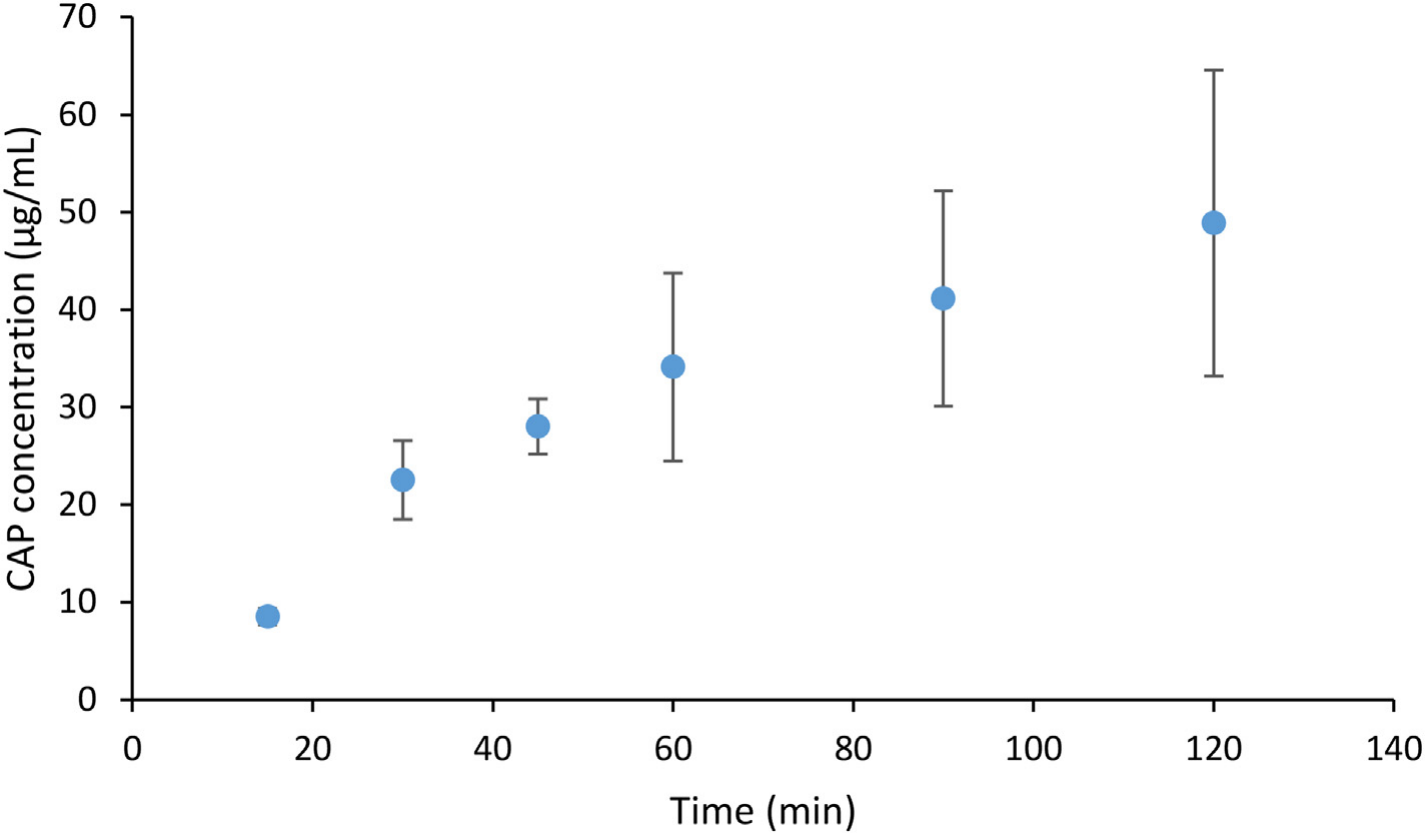
Dissolution of CAP from the 4:6 Lume-CAP granules into 50 mM pH 6.8 phosphate buffer.

The low drug release from the CAP dispersion was unexpected and therefore the polymer release as a function of time was determined, by monitoring the absorbance at 270 nm. At this wavelength both drug and polymer absorb, however, minimal drug is released (around 1–1.5 μg/mL), and hence any absorbance at this wavelength is mainly due to the polymer. The polymer release is shown in Fig. 10, wherein it can be seen that less than 20% of the polymer had dissolved in the medium. If all the polymer dissolved, as expected at this pH, the concentration would be 300 μg/mL. However, the polymer clearly releases to a greater extent than the drug, and thus the release behavior can be described as incongruent, whereby the drug and polymer release at different rates.

**Fig. 11:**
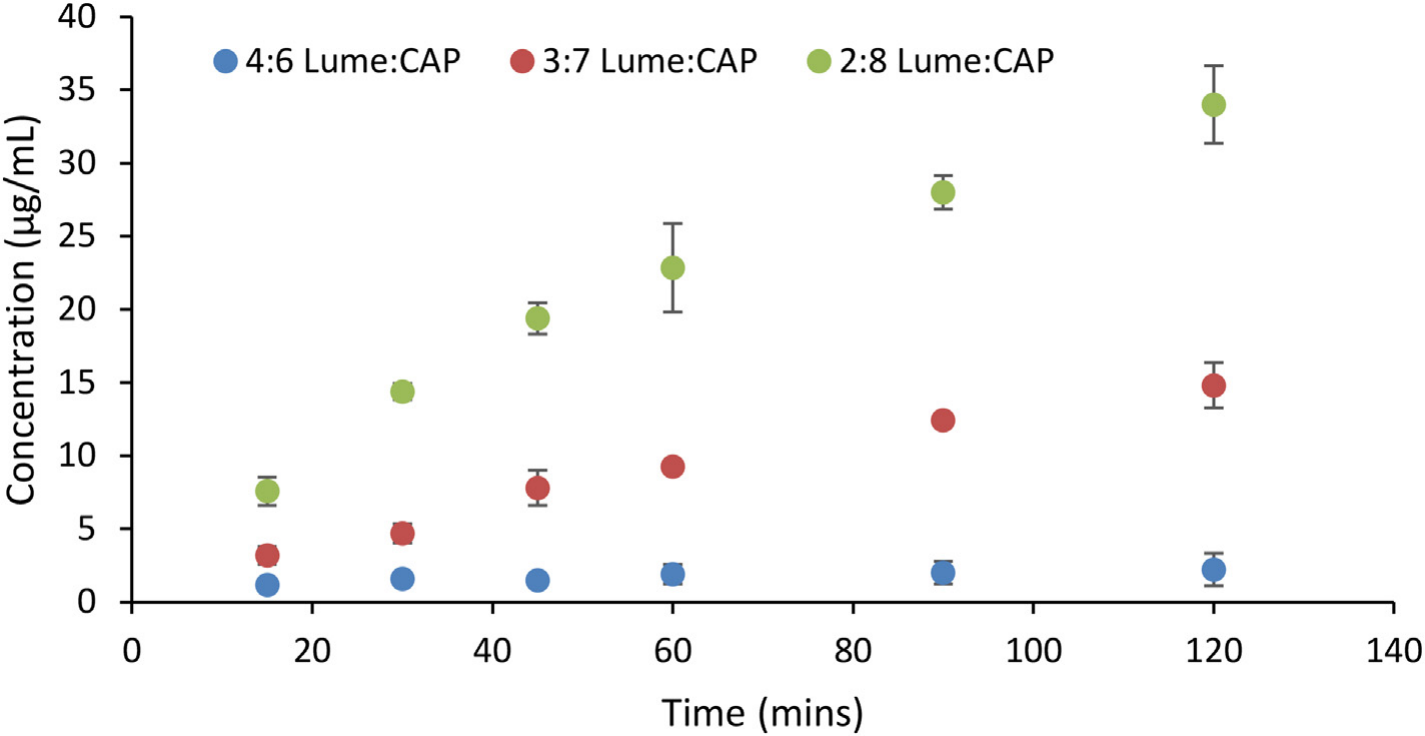
Drug release rate from 20, 30 and 40% drug loaded Lume:CAP dispersions showing faster and more extensive release at a lower drug loading.

To further understand the origin of the poor drug release from CAP dispersions, lower drug loaded dispersions were prepared and their dissolution evaluated (Fig. 11). The drug release increases remarkably as the drug loading is decreased.

## 5 Discussion

In the marketed tablet, lumefantrine is formulated together with artemether. The bioavailability of artemether when given orally is reasonable with the drug being rapidly and reliably absorbed (Teja-Isavadharm et al., 1996). The drug experiences significant first pass metabolism and is converted to pharmacologically active dihydroartemisinin (Lee and Hufford, 1990). The good absorption of artemether, therefore, does not necessitate any efforts to improve the solubility of the drug. In any case, the amorphization of artemether via dispersion with a polymer was not possible at a reasonable drug loading, presumably due to the rigid molecular structure and the lack of H-bonding groups, precluding interaction with polymers. The low melting point (87 °C) also suggests that, even if the drug could be rendered amorphous, the resultant dispersion would likely have a low T_g_ and consequently not be very stable to crystallization. Lumefantrine, on the other hand, had good glass forming ability suggesting it is a good candidate for an amorphous solid dispersion in terms of being able to prepare a dispersion that is stable against crystallization. Lumefantrine bioavailability and pharmacokinetics have also shown to be improved when it is formulated as a solid dispersion (Jain et al., 2017), although the polymer employed in the formulation was not described and no physical stability testing was reported.

Despite the drug having good glass forming ability, the polymer used to form an ASD with lumefantrine clearly has a critical impact on both the ability to produce a completely amorphous formulation, as well as the resistance of that formulation to crystallization upon exposure to high stress storage conditions. Wet granulation of a drug-polymer solution on the excipient powder followed by drying involves much slower solvent evaporation relative to other manufacturing practices such as spray drying, leading to a greater susceptibility to crystallization. Moreover, the rate of solvent evaporation can impact drug-polymer miscibility, with faster evaporation favoring miscibility. Encouragingly, our study clearly shows that the wet granulation approach is a viable manufacturing route as long as the appropriate polymers and drug loading are selected.

The variation in lumefantrine crystallization with different polymers can be largely rationalized based on consideration of drug and polymer chemical structure and properties. First, we consider the neutral polymer, PVPVA. PVPVA is commonly used to form ASDs via hot melt extrusion, and based on the melting point of lumefantrine, this would be a potential manufacturing route for this drug. Indeed, in the bioavailability study of Jain et al. (Jain et al., 2017), the formulations were prepared using this approach. However, this polymer is clearly not effective at inhibiting crystallization, especially under stress storage conditions. PVPVA and lumefantrine have limited opportunities to form specific intermolecular interactions, which, combined with the hygroscopicity of PVPVA and the high Log P of lumefantrine, most likely renders the dispersion susceptible to amorphous phase separation and subsequent crystallization (Rumondor and Taylor, 2010; Rumondor et al., 2011). In contast, the polymers bearing acidic functionalities, can interact more strongly with the weakly basic tertiary amine group of lumefantrine, with the extent of interactions expected to be dependent on the amount of acid groups present in the polymer. Out of the four acidic polymers evaluated, HPMCP, Eudragit L100, and CAP are highly effective, while HPMCAS is a poorer inhibitor. We can probably attribute the diminished performance of HPMCAS to a low mole percentage of carboxylic acid groups (Van Eerdenbrugh and Taylor, 2011), relative to the other acidic polymers (see Table S1), and hence the formation of few strong drug-polymer interactions. The phthalate (acidic bearing moiety) content is between 24 and 30% in HPMCP and between 30 and 36% in CAP (Sheskey et al., 2017) while Eudragit L100 is a 1:1 copolymer of methacrylic acid and methylmethacrylate with a relatively high abundance of carboxylic acid groups. In a previous study with lumefantrine, it was noted that spray dried ASDs with HPMCP and Eudragit L100 showed about 75% protonation (transfer of a proton from the polymer carboxylic acid group to the tertiary nitrogen of the drug, indicating formation of a drug-polymer salt where the polymer is acting as the counterion) when the drug loading was 40%, while the HPMCAS dispersion showed no proton transfer (Song et al., 2016). In the study by Song et al., the varying extent of protonation could not be correlated to either the number of acid moieties on the polymer, or the strength of the acidic group. Instead, steric hindrance and local polymer structure were thought to be important factors. The XPS studies performed herein show that for the sample prepared using solvent evaporation, the extent of protonation varies from 16 to 61%, depending on the polymer used. As seen by Song et al. (Song et al., 2016), there is no apparent correlation between the extent of proton transfer and the number of proton donors present in the polymer. Interestingly, the physical stability of the dispersions correlates with the extent of protonation. The more extensive protonation observed for for HPMCP, CAP and L100 dispersions relative to HPMCAS systems, thus appears to account for the excellent stability of these formulations against crystallization, even when stored at 40 °C/75% RH. Salt formation between drug and an acidic polymer has been reported previously, and was also correlated with improved physical stability to crystallization (Weuts et al., 2005).

Given that the dose of lumefantrine is 120 mg, a high drug loading dispersion is preferable in order to formulate an oral dosage form of reasonable size. The 40 wt% drug loading ASD leads to a total mass of 300 mg of ASD as the intermediate material to be used in the final formulation, which is reasonable. However, it is well known that dissolution performance can be impaired at higher drug loadings (Mosquera-Giraldo et al., 2018; Purohit and Taylor, 2017), and that amorphous drug-rich colloids, thought to be beneficial for oral absorption, may not be formed (Stewart et al., 2017; Wilson et al., 2018). First, we note that all of the dispersions lead to a significant degree of supersaturation. The crystalline solubility was determined to be <80 ng/mL (which was the detection limit for the analytical method), but all ASDs with the acid polymers yielded concentrations of greater than 1 μg/mL, i.e. a degree of supersaturation of at least 20 fold. Second, three of the ASDs also yielded drug-rich nanodroplets in solution indicating that the concentration of the drug in the dissolution medium exceeded the amorphous solubility (2-3 μg/mL). This observation is interesting, as drug-rich nanodroplets are typically only formed from the dissolution of ASDs with lower drug loadings, in the range of 10–20% drug (Indulkar et al., 2017; Jackson et al., 2016; Purohit and Taylor, 2017; Wilson et al., 2018). In this case however, the drug loading cut-off for lumefantrine ASDs which form nanodroplets appears to be much higher.

Relative to the other ASDs formulated with acidic polymers, the dissolution profile of the CAP dispersion was anomolous in that the drug concentration never attained the amorphous solubility and the polymer release was very slow. The IR spectrum and XPS data suggest that CAP forms the most extensive interactions with the drug out of all of the polymers. The reduced release is contrary to previous studies of ASDs where salt formation was observed, where improved release was observed following the formation of ionic interactions (Weuts et al., 2005). Strong interactions could conceivably result in an insoluble drug-polymer complex, causing the observed low drug and polymer release. This type of phenomenon has been observed between PVP and polyacrylic acid in aqueous solution wherein the two soluble polymers form strong hydrogen bonds with each other and precipitate from solution (Paladhi and Singh, 1994). However, much improved dissolution is obtained when the drug loading is reduced to 20% (Fig. 11). This indicates that insoluble complex formation is not the likely explanation. Rather, it is likley that a shift from polymer-controlled to drug-controlled release kinetics has occurred as the drug loading increases (Indulkar et al., 2019). The fact that this drop-off in dissolution rate occurs for CAP at a lower drug loading relative to the other acidic polymers could, however, be related to the stronger drug-polymer interactions observed for this system. Clearly more studies are warranted to better understand this phenomenon.

## 6 Conclusions

Physically stable amorphous solid dispersions of lumefantrine with acidic polymers were successfully prepared using a simple solvent granulation process. Drug release from these granules was found to be fast resulting in the formation of supersaturated solutions and drug-rich nanodroplets. Eudragit L100 appeared to provide the best balance between stability against crystallization and drug release during dissolution, at a high drug loading of 40 wt%, while cellulose acetate phthalate formed strong, ionic interactions with the drug and inhibited drug release. Proton transfer between drug and polymer, leading to formation of ionic interactions and the formation of a drug-polymer salt, contributed to the excellent stability of some of the dispersions against crystallization when stored at high relative humidity conditions. This study highlights the complex interplay between drug loading, drug-polymer interactions, physical stability and release properties.

## Declaration of Competing Interest

The authors declare that they have no known competing financial interests or personal relationships that could have appeared to influence the work reported in this paper.

## Appendix A Supplementary data

Supplementary material Image 1
