# Supplementary material for: Physical stability and release properties of lumefantrine amorphous solid dispersion granules prepared by a simple solvent evaporation approach
Source: Int J Pharm X. 2020 Jul 16;2:100052. doi: 10.1016/j.ijpx.2020.100052 (PMC7390794; doi:10.1016/j.ijpx.2020.100052)
Supplement: Supplementary file 1 — Supplementary material [file mmc1.docx]

Supplementary Information

Physical Stability and Release Properties of Lumefantrine Amorphous Solid Dispersion Granules Prepared by a Simple Solvent Evaporation Approach

Niraj S. Trasi^1,2^, Sonal V. Bhujbal^1^, Dmitry Y. Zemlyanov, Qi (Tony) Zhou^1^, Lynne S. Taylor^1.*^

^1^Department of Industrial and Physical Pharmacy, College of Pharmacy, Purdue University, West Lafayette, Indiana 47907, United States

^2^ Current address- Analytical Development, Celgene Corporation, 556 Morris Avenue, Summit, NJ 07901, United States.

^3^ Birck Nanotechnology Center, Purdue University, West Lafayette, Indiana 47907, United States


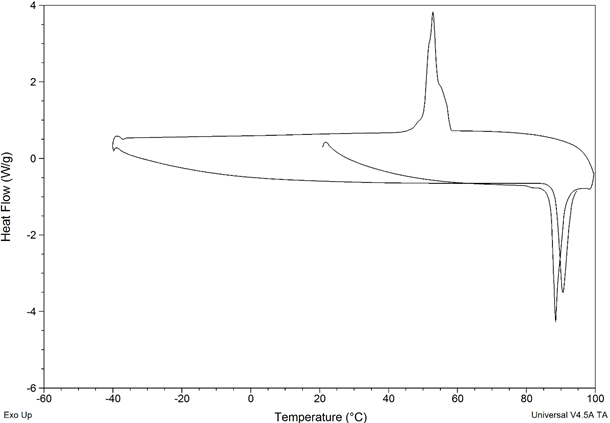


heat 1

heat 2

melting

crystallization

**Figure S1. DSC thermogram of artemether showing the melting endotherm and recrystallization peak.**


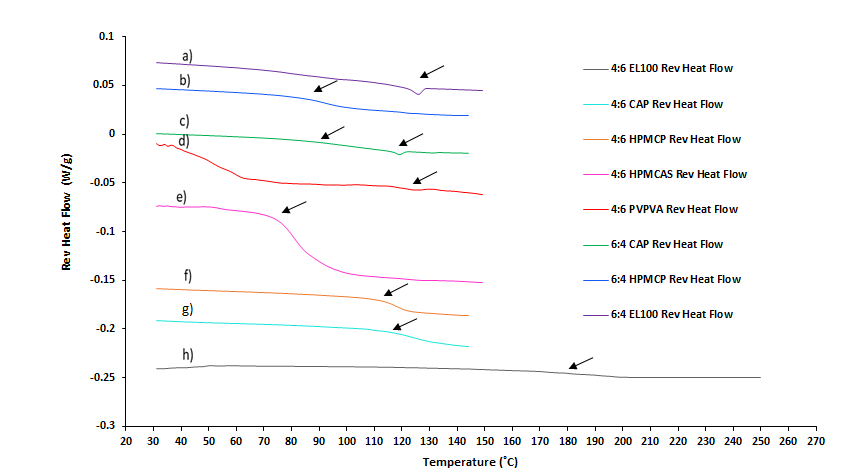


**Figure S2. DSC thermograms of 6:4 Lumefantrine:polymer prepared by rotary evaporation of lumefantrine with (a) Eudragit L100, (b) HPMCP, (c) CAP and, 4:6 lumefantrine:polymer prepared with d) PVPVA, (e) HPMCAS, (f) HPMCP, (g) ) CAP, (h) Eudragit L100. Arrows indicate thermal events, either the glass transition, or the melting of crystalline lumefantrine, where the crystallinity is thought to evolve during the DSC scan. The melting point of crystalline lumefantrine is 129°C, and this value may be lower in the presence of polymer due to melting point depression. DSC scans were not run for 6:4 HPMCAS and PVPVA since these samples crystallized during preparation.**

**Figure S3. Infrared spectrum of crystalline lumefantrine**

**Table 1. Amount of reactive groups per gram of substance for lumefantrine and polymers. Data taken from Song et al.**

| **Compound** | **mmol of functional group per gram of substance** |
| --- | --- |
| **Lumefantrine** | **1.9** |
| **EL100** | **5.8** |
| **CAP** | **2.0** |
| **HPMCAS** | **1.0** |
| **HPMCP** | **2.0** |
| **PVPVA** | **0.0** |

**References**

Song, Y., Zemlyanov, D., Chen, X., Su, Z., Nie, H., Lubach, J.W., Smith, D., Byrn, S., Pinal, R., 2016. Acid-base interactions in amorphous solid dispersions of lumefantrine prepared by spray-drying and hot-melt extrusion using x-ray photoelectron spectroscopy. Int J Pharm 514, 456-464.
